# Access to Essential Medicines and Diagnostic Tests for Cardiovascular Diseases in Maputo City, Mozambique

**DOI:** 10.5334/gh.1186

**Published:** 2023-02-28

**Authors:** Neusa Jessen, Abhishek Sharma, Jennifer Jones, Tangeni Auala, Sainimere Boladuadua, Ahmadou Jingi, Jose Ferrer, Ana Olga Mocumbi

**Affiliations:** 1 World Heart Federation, Geneva, Switzerland; 2 Research Unit of the Department of Medicine, Maputo Central Hospital, Maputo, Mozambique; 3 Faculty of Medicine, Eduardo Mondlane University, Maputo, Mozambique; 4 Department of Global Health, Boston University School of Public Health, Boston, Massachusetts, United States of America; 5 National University of Ireland Galway, Ireland; 6 National Institute for Prevention and Cardiovascular Health, Galway, Ireland; 7 Cardiac Clinic Division of Cardiology, University of Cape Town, Republic of South Africa; 8 Division of Adult Cardiology, Windhoek Central Hospital Complex, Windhoek, Republic of Namibia; 9 School of Medicine, Faculty of Medical and Health Sciences, University of Auckland, New Zealand; 10 University of Yaounde, Cameroon; 11 American Heart Association International, Dallas, United States; 12 Instituto Nacional de Saúde, Ministério da Saúde, Av Eduardo Mondlane, Caixa Postal 264, Maputo, Moçambique

**Keywords:** essential medicines, cardiovascular medicines, cardiovascular diagnostics, Maputo, Mozambique

## Abstract

**Background:**

To tackle the increasing burden of non-communicable diseases (NCDs) and reduce premature cardiovascular (CV) mortality by a third by the year 2030, countries must achieve 80% availability of affordable essential medicines (EMs) and technologies in all health facilities.

**Objectives:**

To evaluate access to EMs and diagnostics for CV diseases in Maputo City, Mozambique.

**Methods:**

Using a modified version of World Health Organization (WHO)/Health Action International (HAI) methodology, we collected data on availability and price of 14 WHO Core EMs and 35 CV EMs in all 6 public-sector hospitals, 6 private-sector hospitals, and 30 private-retail pharmacies. Data on 19 tests and 17 devices were collected from hospitals. Medicine prices were compared with international reference prices (IRPs). Medicines were considered unaffordable if the lowest paid worker had to spend more than one day’s wage to purchase a monthly supply.

**Results:**

Mean availability of CV EMs was lower than that of WHO Core EMs in both public (hospitals: 20.7% vs. 52.6%) and private sectors (retail pharmacies: 21.5% vs. 59.8%; hospitals: 22.2% vs. 50.0%). Mean availability of CV diagnostic tests and devices was lower in public (55.6% and 58.3%, respectively) compared to private sector (89.5% and 91.7%, respectively). Across WHO Core and CV EMs, the median price of lowest priced generic (LPG) and most sold generic (MSG) versions were 4.43 and 3.20 times the IRP, respectively. Relative to the IRP, median price of CV medicines was higher than that of Core EMs (LPG: 4.51 vs. 2.93). The lowest paid worker would spend 14.0 to 17.8 days’ wage monthly to undergo secondary prevention.

**Conclusion:**

Access to CV EMs is limited in Maputo City owing to low availability and poor affordability. Public-sector hospitals are not well equipped with essential CV diagnostics. This data could inform evidence-based policies for improving access to CV care in Mozambique.

## Introduction

Globally, cardiovascular diseases (CVD) continue to be the leading cause of mortality, accounting for estimated 17.6 million deaths (i.e., 31% of all deaths) annually [1]. Low- and middle-income countries (LMICs) are most affected and account for over 80% of global CVD mortality [2]. This increasing disease risk is driven by rising levels of lifestyle-related risk factors and the inability of healthcare systems to provide adequate CVD risk prevention, early detection and treatment [2].

In 2012, the United Nations (UN) member states adopted a global commitment to reduce premature non-communicable diseases (NCDs) burden by a third by the year 2030 [3]. Subsequently, various international agencies have promulgated these goals in their agendas, including the World Health Organization (WHO)’s 2013–2020 Global Action Plan [4], World Heart Federation (WHF)’s 25 × 25 vision [5], and UN 2030 Sustainable Development Goals [3]. However, to achieve these goals, equitable and affordable access to essential medicines (EMs) and technologies for managing CVD is critical. A 2001 resolution (WHA 54.11) by the member states of the WHO called for a methodology to monitor medicine prices to improve access. In response, the WHO/Health Action International (WHO/HAI) methodology was developed in 2003 to monitor medicine availability, consumer prices and affordability in a reproducible way, allowing international comparisons over time [6]. Realizing that medicine availability and affordability are key components of patient access, the WHO medium term strategic plan 2008–2013 defines a) global and national targets of 80% for availability for EMs in health facilities in all sectors; and that b) no patient should pay more than four times the international reference price (IRP) for a given medicine [7]. While governments should be procuring medicines on the international market as close to IRPs, private-sector patient prices have to take into account additional costs (such as taxes, tariffs, margins) in the pharmaceutical supply chain [7].

Mozambique – a low-income, sub-Saharan African country – faces a high NCD burden that accounts for 28% deaths, of which 12% are due to CVDs [8]. Mozambique is one of the poorest countries worldwide [9], less urbanized than other countries in Southern Africa [10,11,12] and highly dependent on donor aid to implement its key health programs [13] and research. Patients predominantly seek subsidized healthcare in the public sector, wherein hospital pharmacies dispense medicines at a charge of 5MTs (0.08$) per prescription. However, these public-sector health facilities are over-burdened due to high caseload, limited resources and infrastructure, poor management, and inefficient medicine supply. This often forces patients, especially those in urban areas, to seek healthcare in the private sector through out-of-pocket (OPP) payments. Although NCDs are now included in several important government documents such as the Government Five Year Plan (2015–2019) and the Health Sector Strategic Plan (PESS 2014–2019), and, a Policy framework for inter-sectorial prevention and control of NCDs was included in the Government of Mozambique 2017–2020 United Nation Development Assistance Framework (UNDAF), very little survey data exists to provide a baseline measure of access to essential CVD medicines and diagnostics in Mozambique.

Multiple surveys have highlighted variations in medicine availability and prices across regions, therapeutic categories, and health sectors. An analysis of surveys conducted during 2008–2015 found that very few medicines met WHO’s 80% availability target in LMICs [14]. However, such available data from over 100 other surveys provide a limited perspective in terms of CVD medicines access. The most commonly surveyed medicine basket includes a limited number of essential CVD medicines and thus offers a limited perspective on access to CVD treatments. Furthermore, there is a lack of data on access to CV diagnostics in Mozambique. This information would be invaluable to guide decisions and policies aimed at addressing the unique CVD profile in LMICs, that is often linked to poverty and/or to uncontrolled endemic infections [8]. Therefore, we conducted a representative survey and evaluated access (i.e., availability, prices and affordability) to a comprehensive list of essential CV medicines and diagnostics needed to treat patients with CVD.

## Methods

### Study design

Using a modified version of WHO/HAI methodology [15], we conducted a cross-sectional survey to assess the availability, price and affordability of essential CVD medicines, diagnostic tests, and devices (some for diagnostic and some for non-pharmacologic treatment) in the public and private healthcare sectors in Maputo City, Mozambique. While standard WHO/HAI surveys are usually limited to a pre-defined list of core global medicines along with medicines selected by the investigator, we also surveyed the availability and prices of CVD diagnostics in public and private sector hospitals.

### Sampling

#### Setting and survey facilities

**Figure 1:**
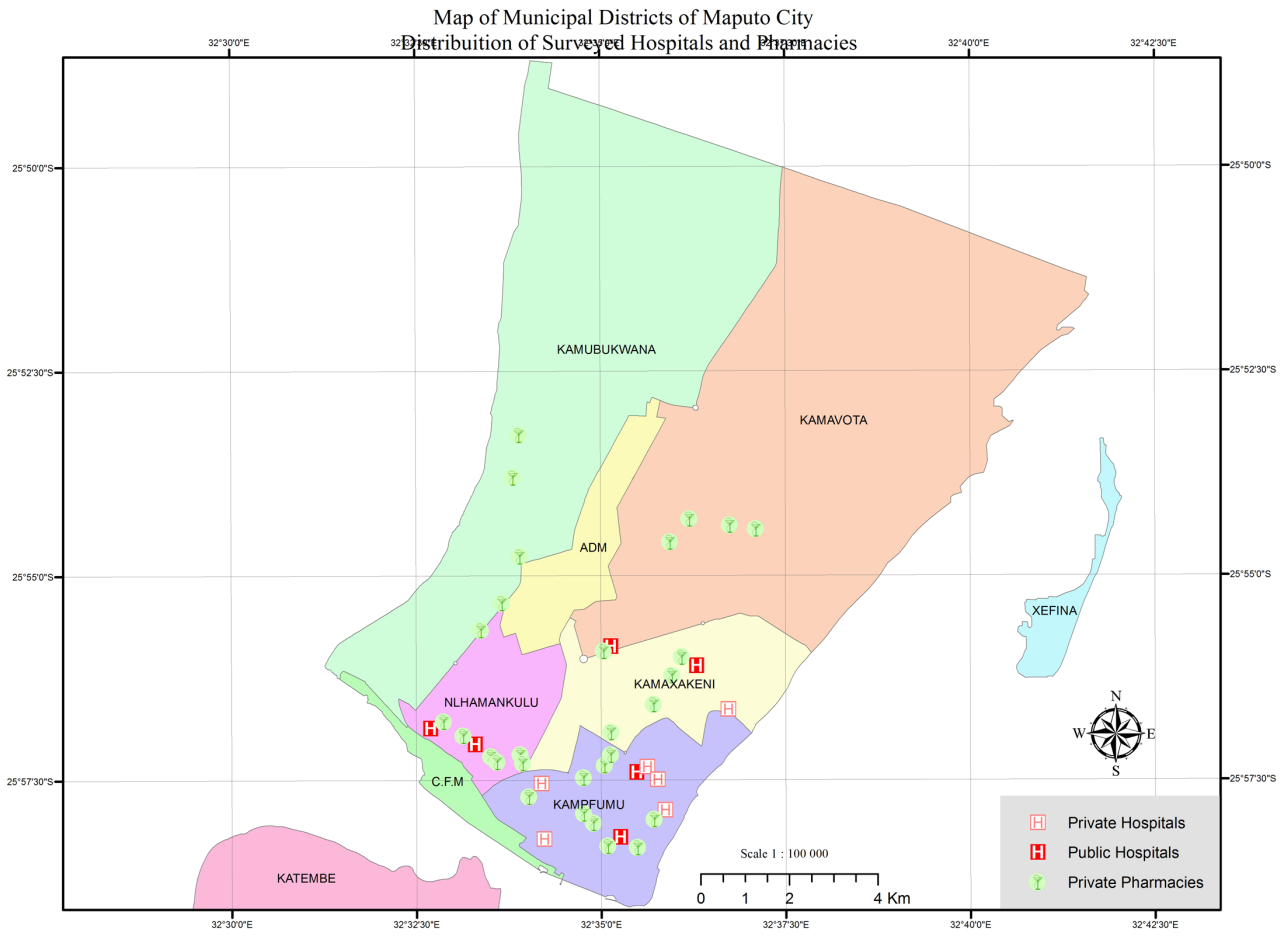
Map of the survey area and location of the surveyed facilities in Maputo city, Mozambique.

Maputo City is the capital and the main urban center in Mozambique. It has a population of over 1.2 million [16] distributed in its seven administrative areas (districts). We selected the major urban administrative area (KaMpfumo district, where the central hospital – tertiary level – is located), and we also included four additional districts in Maputo city, all of which were within three hours of travel by public transport from KaMpfumo district, performing a total of five mainland survey districts. We selected the public-sector tertiary level hospital and one secondary level hospital in each survey district (‘survey anchors’). For districts that did not have public hospitals of the desired level, we selected the nearest hospital located in a neighboring district as the survey anchor. This sampling methodology led to the inclusion of all public-sector hospitals in Maputo City: the tertiary level and the five first-referral hospitals, where public-sector medicine and diagnostic price data was collected. We then selected five private-sector retail pharmacies around each of the six survey anchors, resulting in a total of 30 pharmacies. We also selected six private-sector hospitals located throughout the neighbourhoods of KaMpfumo district to evaluate access to CVD diagnostics. Of note, this included all the private hospitals in the district (Figure 1).

#### Survey medicines and diagnostics

In the absence of local CVD guidelines, we reviewed relevant guiding documents – including the WHO Model Essential Medicines List (EML), the national EML, the Priority Life-Saving Medicines for Women and Children document [17] – and sought local expert-opinion (pediatric and adult cardiologists) to develop a comprehensive list of survey CVD medicines, diagnostic tests and devices. These commodities were deemed important for delivering rational CVD care in both the public and private healthcare sectors. The final list of survey items contained 14 medicines from the WHO/HAI core global medicines list, 35 CVD medicines (total 41 dosage forms), 19 diagnostic tests and 17 devices (Tables 1 and 2).

**Table 1:** Availability of surveyed essential medicines in Maputo city, Mozambique.


		PRIVATE-SECTOR		PUBLIC-SECTOR HOSPITAL PHARMACIES [N = 6]

		RETAIL PHARMACIES [N = 30]	HOSPITAL PHARMACIES [N=6]	

**Core Essential Medicines**				

1	Amitriptyline 25 mg	36.7% (n = 11)	83.3%	83.3% (n = 5)

2	Amoxicillin 500 mg	100.0% (n = 30)	100.0%	100.0% (n = 6)

3	Atenolol 50 mg	70.0% (n = 21)	33.3%	16.7% (n = 1)

4	Captopril 25 mg	20.0% (n = 6)	0.0%	0.0% (n = 0)

5	Ceftriaxone Inj 1 g/via	46.7% (n = 14)	50.0%	50.0% (n = 3)

6	Ciprofloxacin 500 mg	80.0% (n = 24)	66.7%	66.7% (n = 4)

7	Co-trimoxazole suspension 8+40 mg/ml	50.0% (n = 15)	66.7%	66.7% (n = 4)

8	Diazepam 5 mg	3.3% (n = 1)	33.3%	33.3% (n = 2)

9	Diclofenac 50 mg	100.0% (n = 30)	83.3%	83.3% (n = 5)

10	Glibenclamide 5 mg	90.0% (n = 27)	66.7%	66.7% (n = 4)

11	Omeprazole 20 mg	60.0% (n = 18)	16.7%	**

12	Paracetamol suspension 24 mg/ml	56.7% (n = 17)	16.7%	33.3% (n = 2)

13	Salbutamol inhaler 100 mcg/dose	56.7% (n = 17)	33.3%	33.3% (n = 2)

14	Simvastatin 20 mg	70.0% (n = 21)	50.0%	50.0% (n = 3)

**CVD Essential Medicines**				

1	Bisoprolol 5mg Tab/cap	43.3% (n = 13)	16.7%	16.7% (n = 1)

2	Glyceryl trinitrate (sublingual) 0.5 mg Tab/cap	0.0% (n = 0)	0.0%	0.0% (n = 0)

3	Isosorbid dinitrate (sublingual) 5 mg Tab/cap	0.0% (n = 0)	0.0% (n = 0)	0.0% (n = 0)

4	Digoxin 0.05 mg/ml solution	0.0% (n = 0)	0.0% (n = 0)	0.0% (n = 0)

5	Lidocaine 200 mg/ml in 5 ml vial	3.3% (n = 1)	0.0% (n = 0)	0.0% (n = 0)

6	Verapamil (hydrochloride) 40 mg Tab/cap	0.0% (n = 0)	0.0% (n = 0)	0.0% (n = 0)

7.a	Amiodarone 100 mg Tab/cap	0.0% (n = 0)	0.0% (n = 0)	0.0% (n = 0)

7.b	Amiodarone 50 mg/ml in 3 ml Ampoule	0.0% (n = 0)	16.7% (n = 1)	16.7% (n = 1)

8	Amlodipine maleate 5 mg Tab/cap	70.0% (n = 21)	33.3% (n = 2)	16.7% (n = 1)

9	Enalapril (as hydrogen maleate) 5 mg Tab/cap	56.7% (n = 17)	33.3% (n = 2)	16.7% (n = 1)

10.a	Hydralazine, powder for injection 20 mg Ampoule	0.0% (n = 0)	33.3% (n = 2)	33.3% (n = 2)

10.b	Hydralazine 25 mg Tab/cap	0.0% (n = 0)	50.0% (n = 3)	50.0% (n = 3)

11	Hydrochlorothiazide 25 mg Tab/cap	3.3% (n = 1)	16.7% (n = 1)	16.7% (n = 1)

12	Methyldopa 250 mg Tab/cap	90.0% (n = 27)	50.0% (n = 3)	50.0% (n = 3)

13	Losartan 50 mg Tab/cap	36.7% (n = 11)	0.0% (n = 0)	0.0% (n = 0)

14.a	Furosemide 40 mg Tab/cap	90.0% (n = 27)	100.0% (n = 6)	100.0% (n = 6)

14.b	Furosemide 10 mg/ml in 2ml ampoule	0.0% (n = 0)	50.0% (n = 3)	50.0% (n = 3)

14.c	Furosemide 20 mg/5ml vial	0.0% (n = 0)	16.7% (n = 1)	16.7% (n = 1)

15	Spironolactone Tab/cap	70.0% (n = 21)	33.3% (n = 2)	33.3% (n = 2)

16	Dopamine vial	0.0% (n = 0)	0.0% (n = 0)	0.0% (n = 0)

17	Acetylsalicylic acid Tab/cap	40.0% (n = 12)	50.0% (n = 3)	50.0% (n = 3)

18	Clopidogrel Tab/cap	26.7% (n = 8)	16.7% (n = 1)	16.7% (n = 1)

19	Streptokinase vial	0.0% (n = 0)	0.0% (n = 0)	0.0% (n = 0)

20	Gliclazide (controlled release) Tab/cap	20.0% (n = 6)	16.7% (n = 1)	16.7% (n = 1)

21	Glucagon injection	0.0% (n = 0)	0.0% (n = 0)	0.0% (n = 0)

22	Metformin (hydrochloride) Tab/cap	90.0% (n = 27)	16.7% (n = 1)	16.7% (n = 1)

23	Benzathine benzyl penicillin vial	33.3% (n = 10)	50.0% (n = 3)	50.0% (n = 3)

24	Heparin vial	0.0% (n = 0)	0.0% (n = 0)	0.0% (n = 0)

25	Warfarin Tab/cap	30.0% (n = 1)	33.3% (n = 2)	33.3% (n = 2)

26	Morphine ampoule	0.0% (n = 0)	16.7% (n = 1)	33.3% (n = 2)

27	Phenoxymethyl penicillin 250 mg Tab/cap	0.0% (n = 0)	33.3% (n = 2)	0.0% (n = 0)

28	Erythromycin Tab/cap	0.0% (n = 0)	50.0% (n = 3)	50.0% (n = 3)

29	Nifedipine retard Tab/cap	70.0% (n = 21)	83.3% (n = 5)	83.3% (n = 5)

30	Soluble insulin vial 100 IU/ml	0.0% (n = 0)	0.0% (n = 0)	83.3% (n = 5)

31	Adrenaline vial	0.0% (n = 0)	16.7% (n = 1)	33.3% (n = 2)

32	Sodium Nitroprusside, powder for infusion ampoule	0.0% (n = 0)	0.0% (n = 0)	0.0% (n = 0)

33.a	Metoclopramide (hydrochloride) ampoule	6.7% (n = 2)	33.3% (n = 2)	16.7% (n=1)

33.b	Metoclopramide, Oral liquid: 5 mg/5 mL	3.3% (n = 1)	0.0% (n = 0)	16.7% (n=1)

33.c	Metoclopramide, Solid oral: 10 mg (hydrochloride)	36.7% (n = 11)	0.0% (n = 0)	0.0% (n= 0)

34	Digoxin Tab/cap	46.7% (n = 14)	**	**

35	Phenoxymethyl penicillin 500 mg Tab/cap	33.3% (n = 10)	**	**

	**Overall availability of Core Essential Medicines**			

	**• Mean (SD)**	59.8% (28.2%)	50.0% (29.2%)	52.6% (28.7%)

	**• Median (IQR)**	56.7% (47.5%, 77.5%)	50.0% (33.3%, 66.7%)	50.0% (33.3%, 66.7%)

	**• [min, max]**	[3.3%, 100.0%]	[0.0%, 100.0%]	[0.0%, 100.0%]

	**Overall availability of CVD Medicines**			

	**• Mean availability**	21.5% (29.3%)	22.2% (24.6%)	20.7% (25.8%)

	**• Median (IQR)**	3.3% (0.0%, 36.7%)	16.7% (0.0%, 33.3%)	16.7% (0.0%, 33.3%)

	**• [min, max]**	[0.0%, 90.0%]	[0.0%, 33.3%]	[0.0%, 100.0%]

	**ALL Medicines**	30.6% (33.2%)	29.6% (28.4%)	28.4% (29.6%)

		20.0% (0.0%, 56.7%)	16.7% (0.0%, 50.0%)	16.7% (0.0%, 100.0%)

		[0.0%, 100.0%]	[0.0%, 100.0%]	[0.0%, 100.0%]


** Missing data (medicines not surveyed because they were included in the survey list after the data collection in those facilities was completed).

**Table 2:** Availability and price of CVD diagnostics in Maputo city, Mozambique.


	AVAILABILITY (%)				PRICE (MTN|USD)
	
	PUBLIC-SECTOR HOSPITALS		PRIVATE-SECTOR HOSPITALS		PRIVATE SECTOR
	
	IN GENERAL	AT THE TIME OF SURVEY	IN GENERAL	AT THE TIME OF SURVEY	

**Diagnostic tests**					

Glycaemia	100%	100%	100.0%	100.0%	304.5|4.99

Creatinine	100%	100%	100.0%	100.0%	300.0|4.92

Urea	100%	83.3%	100.0%	83.3%	266.0|4.36

Total cholesterol	83.3%	50.0%	100.0%	100.0%	437.5|7.17

HDL cholesterol	66.7%	50.0%	100.0%	100.0%	684.0|11.21

LDL cholesterol	50.0%	0.0%	100.0%	100.0%	671.0|11.00

Triglyceride	83.3%	83.3%	100.0%	100.0%	657.0|10.77

Proteinuria	83.3%	83.3%	83.3%	83.3%	349.5|5.73

Natremia	50.3%	33.3%	100.0%	100.0%	336.0|5.51

Kalemia	100.0%	33.3%	100.0%	100.0%	336.0|5.51

HbA1c	16.7%	0.0%	83.3%	83.3%	1373.0|22.51

Uric acid	83.3%	83.3%	100.0%	83.3%	336.0|5.51

Full blood count	100.0%	100.0%	100.0%	100.0%	730.0|11.97

ESR	100.0%	83.3%	83.3%	83.3%	230.0|3.77

Troponin	16.7%	16.7%	50.0%	50.0%	1496.0|24.52

ASO	66.7%	33.3%	66.7%	66.7%	496.0|8.13

Electrocardiogram	33.3%	33.3%	100.0%	100.0%	900.0|14.75

Echodiogram	33.3%	33.3%	66.7%	66.7%	3500.0|57.38

Chest X-ray (radiography facility)	83.3%	83.3%	100.0%	100.0%	1416.0|23.21

**Mean**	**73.7%**	**55.6%**	**91.2%**	**89.5%**	

**Median [min, max]**	**83.3% [16.7%, 100.0%]**	**50.0% [0.0%, 100.0%]**	**100.0% [50.0%, 100.0%]**	**100.0% [50.0%, 100.0%]**	

**Diagnostic devices**					

Thermometer	100.0%	100.0%	100.0%	100.0%	

Electrocardiograph	33.3%	33.3%	100.0%	100.0%	

Weighing scale/machine	100.0%	100.0%	100.0%	100.0%	

Sphygmomanometer	83.3%	66.7%	100.0%	100.0%	

Stethoscope	100.0%	100.0%	100.0%	100.0%	

Pulse oximeter	66.7%	66.7%	100.0%	100.0%	

Spacer for inhalers	83.3%	83.3%	100.0%	100.0%	

Glucometer	100.0%	83.3%	100.0%	100.0%	

Peak flow meter	0.0%	0.0%	66.7%	66.7%	

Blood glucose test strips	50.0%	50.0%	100.0%	100.0%	

Urine protein test strips	16.7%	16.7%	66.7%	66.7%	

Urine ketone test strips	0.0%	0.0%	66.7%	66.7%	

**Mean availability**	**61.1%**	**58.3%**	**91.7%**	**91.7%**	

**Median [min, max]**	**75.0% [0.0%, 100%]**	**66.7% [0.0%, 100.0%]**	**100.0% [66.7%, 100.0%]**	**100.0% [66.7%, 100.0%]**	

**Additional diagnostic and treatment devices**					

Nebulizer	66.7%	66.7%	100.0%	100.0%	

Troponin test strips	0.0%	0.0%	50.0%	50.0%	

Urine albuminuria test strips	0.0%	0.0%	50.0%	50.0%	

Tuning fork	0.0%	0.0%	0.0%	0.0%	

Defibrillator	50.0%	50.0%	100.0%	100.0%	

**Mean availability**	**23.3%**	**23.3%**	**60.0%**	**60.0%**	

**Median [min, max]**	**0.0% [0.0%, 66.7%]**	**0.0% [0.0%, 66.7%]**	**50.0% [0.0%, 100.0%]**	**50.0% [0.0%, 100.0%]**	


CVD, cardiovascular disease; ESR, Erythrocyte sedimentation rate; ASO, antistreptolysin O titer.

### Data collection and analysis

After prior notification, data collectors visited the selected hospitals and private retail pharmacies in March–April 2018. Upon physical inspection and using a standardized survey form, data collectors obtained information on availability as well as the consumer prices of the lowest-priced generic (LPG) and most sold generic (MSG) versions of the survey medicines, that were in stock on the day of survey. Data were also collected for the survey CVD diagnostic tests.

We report the availability of EMs and diagnostics as the percentage of surveyed facilities – in both the public and private sectors – where a given medicine, diagnostic test or device was found on the day of the survey. The overall availability is summarized as ‘mean availability’. To facilitate international comparisons, we calculated medicine-specific median price ratios (MPRs) when consumer price data were available from at least four facilities. The MPR refers to the ratio of a medicine’s local consumer unit price (across pharmacies) as compared to the 2015 Management Sciences for Health (MSH) unit international reference price (IRPs) [18]. We report median consumer prices for the survey diagnostic tests and devices. We conducted an affordability analysis as per WHO/HAI methodology, where a chronic disease medication is considered unaffordable if the lowest paid worker (monthly wage: MTN 4,063; USD $61) must spend >1 day’s wage to purchase a monthly supply.

### Ethical approval

This project, like earlier WHO/HAI surveys, required the collection and analysis of only the availability and prices of CVD medicines and diagnostics. This data is publicly available and was analyzed. We obtained permission from Mozambique’s Ministry of Health to comply with local ethical and legal requirements. Informed consent to use data, on the basis of anonymity, was obtained from the pharmacy and hospital staff.

## Results

### Availability

#### Availability of Core and CVD Essential Medicines

Table 1 summarizes the mean availability of WHO EMs – stratified by medicines group and health sector.

In the public-sector hospital pharmacies, the overall mean availability of all surveyed medicines (Core and CVD medicines combined) was 28.4%. Mean availability of CVD EMs (20.7%) was lower than that of the WHO Core EMs (52.6%). Three CVD medicines, that is, furosemide 40 mg, nifedipine 30mg, and soluble insulin, were available in over 80% of surveyed facilities. One-third (i.e., 14 out of 41 surveyed medicine dosage forms) were not available in any strength or dosage form at any public-sector facility, and these included digoxin, isosorbide dinitrate, losartan, verapamil and phenoxymethyl penicillin.

In the private sector, the mean availability of all EMs (Core and CVD medicines combined) was 30.6% and 29.6% in retail and hospital pharmacies, respectively. Mean availability of CVD medicines (retail: 21.5%; hospital: 22.2%) was lower than that of core EMs (retail: 59.8%; hospital: 50.0%).

In the private-sector retail pharmacies, over a third of the 14 Core EMs – that is, amitriptyline 25 mg, captopril 25 mg, diazepam 5mg, ceftriaxone 1gm vial, and co-trimoxazole suspension 8+40 mg/ml – were available in ≤50% surveyed retail pharmacies.

Of the 41 CVD EMs (inclusive of dosage forms of same medicine), 50% were not found in any private-sector retail pharmacies. This included amiodarone, hydralazine, insulin, glucagon injection, erythromycin and phenoxy methypenicillin. This proportion was even lower in the private-sector hospitals: 57% (8 out of 14) core medicines and 95% (37 out of 39) CVD medicine dosage forms were available in ≤50% surveyed facilities.

#### Availability of CVD diagnostic test

The mean availability of the CVD diagnostic tests was 73.7% and 91.2%, respectively, in the public- and private-sector hospitals. However, on the day of survey, these diagnostic tests were in fact available (i.e., offered/administered to patients) in fewer public (55.6%) and private sector (89.5%) hospitals. This occurred because although the hospital had capacity to perform the tests, the reagents for the tests were not available. Diagnostic tests for glycaemia, creatinine and full blood count were usually offered and were available on the day of survey in all the public-sector hospitals, while LDL-cholesterol and HbA1c tests were not available in any public hospital on the day of survey. Nearly all the surveyed diagnostic tests (except troponin, ASO and echocardiogram) were offered and were available on the day of survey in 80% of the private-sector hospitals.

Mean availability of the surveyed diagnostic devices needed to provide basic CVD care was 61.1% in public hospitals and 91.7% in private sector hospitals. In public-sector hospitals, electrocardiogram, peak flow meter, tuning fork, and strips for urine ketone, urine protein, urine albuminuria and troponin tests were available in less than 35% of the facilities. In the private-sector hospitals, nearly all the diagnostic devices (except troponin and urine albuminuria test strips and tuning fork) were available in over 75% facilities. See Table 2.

### Price

#### Prices of Core and CVD essential medicines

Table 3 summarizes the median unit consumer price and MPR – stratified by medicine version – of the surveyed core and CVD EMs in the private-sector retail pharmacies. Overall, across all medicines (Core and CVD EMs combined), the LPG and MSG versions were 4.43 and 3.20 times the MSH IRPs, respectively. The median MPR of the surveyed CVD medicines was higher [LPG: 4.51 (range: 0.98–58.09); MSG: 5.37 (range: 2.05–58.09)] than that of the Core EMs [LPG: 2.93 (range: 1.67–11.01); MSG: 2.93 (1.67–13.02)].

**Table 3:** Summary of median price ratios (MPRs) for Core and CVD essential medicines in the private retail sector in Maputo region, Mozambique.


	PRIVATE-SECTOR RETAIL PHARMACIES	

	LOWEST PRICED GENERIC	MOST SOLD GENERIC

**Core Essential medicines**		

Median MPR	2.93	2.93

Minimum, Maximum MPR	1.67, 11.01	1.67, 13.02

**CVD Essential Medicines**		

Median MPR	4.51	5.37

Minimum, Maximum MPR	0.98, 58.09	2.05, 58.09

**Overall (All medicines)**		

Median MPR	4.43	3.20

Minimum, Maximum MPR	0.98, 58.09	1.67, 58.09


The core medicines whose LPG versions were higher than four times the IRPs included amitriptyline 25 mg, atenolol 50 mg, ceftriaxone inj 1g/vial, diazepam 5mg, and simvastatin 20 mg. The CVD medicines for which LPG versions was over four times the IRP included amlodipine maleate 5mg, enalapril 5 mg, spironolactone 25 mg, acetylsalicylic acid 100 mg, clopidogrel 75 mg, glicazide (controlled release) 60 mg, benzathine benzyl penicillin 2.4 mega units Injection, metoclopramide solid oral: 10 mg (hydrochloride), digoxin 0.25 mg, and phenoxymethyl penicillin 500 mg. See Appendix Table S2 for details.

Analysis of median unit consumer price and MPR was not possible for the Public Sector because consumers do not pay for each medicine in this sector; a fix cost (5 MTN = 0.08 USD) is charged for each prescription, independent of the number of medicines prescribed.

Originator brand of the surveyed medicines were not available in public hospital pharmacies and rarely found in private sector, both in retail pharmacies (only Adalat Bayer and Aspirin Bayer were available, at a price much more expensive than the generic version) and in hospital pharmacies (originator brand of vallium, lanoxin and plavix were the only found).

#### Price of CVD Diagnostic tests

In the private-sector hospitals, the median unit price of the surveyed diagnostic tests ranged from MTN 230.0 (ESR) to MTN 3500.0 (echocardiogram). For 11 out of 19 surveyed routine diagnostic tests, the median unit consumer price was more than MTN 400 (Table 2).

### Affordability

**Figure 2:**
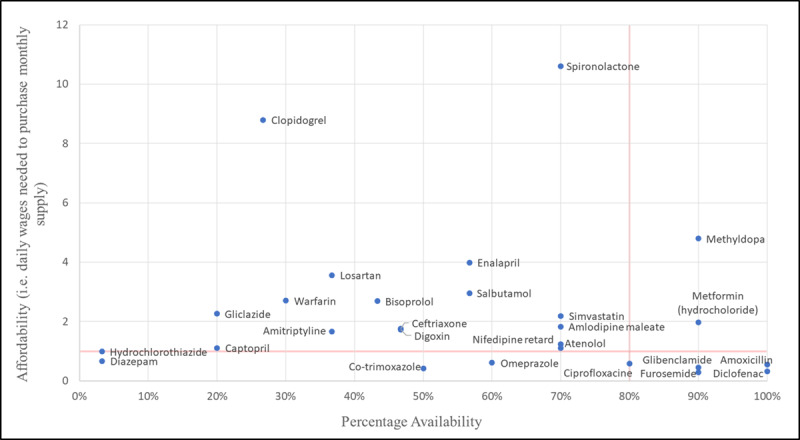
Availability and affordability of selected essential medicines in the private sector.

In our private-sector medicine affordability analysis, we found that the lowest-paid unskilled worker would have to spend more than one day’s wage to out-of-pocket purchase monthly supply of most CVD EMs. See Figure 2 and Appendix Table S2.

Table 4 shows the estimated monthly costs of managing different cardiovascular risk profiles in Maputo’s private sector in terms of number of lowest daily wages. At the lowest CVD risk, requiring only annual risk monitoring, the costs for such management would be 2–4 days’ wage per month. As the risk profiles increase for primary prevention, the estimated cost increases. The ranges are based on the least and most expensive of the various medicines listed. Thus, a lowest paid worker with high CVD-risk who undergoes private-sector interventions for primary prevention would be spending average 2–15 days’ wage each month on these interventions. If this person were undergoing clinical CVD interventions as secondary prevention, the expenditure would be 14–18 days’ wage.

**Table 4:** Estimated monthly costs of managing cardiovascular risk profile in Mozambique’s private sector.


PREVENTION	RISK	INTERVENTION	COST OF MEDICINES (USD | NO. OF DAYS’ WAGES)	COST OF TESTS (USD | NO. OF DAYS’ WAGES)	TOTAL COST (USD | NO. OF DAYS’ WAGES)

**Primary**	<10%	Lifestyle changes + 12-monthly risk monitoring	N/A	4.44 | 2.00	4.44 | 2.00

	10–20%	Lifestyle changes + 6-monthly risk monitoring	N/A	8.88 | 4.00	8.88 | 4.00

	20–30%	Statin^a^ + one antihypertensive^b^ + 6-monthly risk monitoring	7.32–12.79 | 3.3–5.8	8.88 | 4.00	16.2–21.67 | 7.30–9.76

	≥30%	Statin^a^ + one antihypertensive^b^ + aspirin + 3-monthly risk monitoring	10.98–16.45 | 5.0–7.4	17.78 | 8.01	28.76–34.23 | 12.95–15.42

**Secondary**		ß-blocker^c^ + ACE Inhibitor^d^ + statin ^a^ + aspirin + 3-monthly risk monitoring	13.44–21.79 | 6.1–9.8	17.78 | 8.01	31.22–39.57 | 14.06–17.82


Risk monitoring (lipid profile, fasting blood sugar and proteinuria) would cost USD 53.3, that is, 24.01 days’ wages. From these values, we calculated 12-monthly, 6-monthly and 3-monthly risk monitoring costs. Lowest daily wage for unskilled workers in Mozambique at the time of survey was USD 2.22. ^a^ Includes simvastatin; ^b^ Includes amlodipine, nifedipine, hydrochlorothiazide, captopril and enalapril; ^c^ Includes atenolol and bisoprolol; ^d^ Includes captopril and enalapril.

## Discussion

To our best knowledge, this is the first study to evaluate availability and affordability of both essential diagnostics and medicines needed to treat CVD in Mozambique. Overall, we found that availability of essential cardiovascular diagnostics and medicines in both the public and the private sector are low and do not meet WHO’s 80% availability target. While the availability of core EMs (medicines, identified by the WHO/HAI methodology, as indicators of medicine access situation) was just above 50% in both public and private sectors, the mean availability of CVD EMs was even lower, at <25%. The mean availability of CVD EMs was lower in the public hospitals compared to the private sector, thereby compromising the benefit of affordability offered by government subsidized healthcare. For medicines not available in the public sector, patients would have to spend between 3–100% of their monthly wage (approximately 1–30 work days) to purchase a month’s supply of a medicine. Risk monitoring (including lipid profile, fasting blood sugar, and proteinuria) for individuals with low CVD risk would cost USD 4.44, that is, equivalent 2 days’ wages.

These results reveal lower availability of medicines compared to that reported from urban areas in Cameroon (36.4 to 59.1%) [23] and from Nepal (around 50%) [19]. Regarding affordability, the cost of managing individuals with high cardiovascular risk was comparable to that found in Cameroon (USD 28.8 to 34.2 in Mozambique versus 30.7 in Cameroon); however, medicines were less affordable in Mozambique compared to Nepal where on average, the lowest-paid worker would spend 1.03 (public-sector) and 1.26 (private-sector) days’ wages to purchase a monthly supply [19].

Our comprehensive list of survey medicines and diagnostics has the potential to be upscaled, with adaptation as per a given country’s CVD profile, and to inform decision making for improving access to CVD care. This modified list of survey medicine addresses the needs for endemic CVD related to poverty affecting predominantly children and young adults (such as uncorrected congenital heart diseases, rheumatic heart disease, neglected cardiomyopathies, nutritional and infectious diseases), and women of reproductive age [8,20,21]. Its use shows low availability of paediatric formulations and safe drugs for peripartum period and reveals how unaffordable the CVD management is in this resource poor setting.

The present study was performed in Maputo City, the economic center of Mozambique, where referral hospitals with the highest concentration of specialized health services countrywide (including cardiac catheterization and open heart surgery at Hospital Central de Maputo) and the highest availability of drugs are found [22]. As such, we believe that our results represent the best case scenario, and CV medicines and diagnostics are less available and affordable in the rest the country [23].

Medicine availability was higher in the private retail pharmacies when compared to public facilities, and originator brands were only found in the private sector, where patients would need to pay out-of-pocket (OOP) more than four times the maximum acceptable IRP according to the WHO [7]. This represents a high burden of health expenditure in the household’s budget, as previously reported in a national census [24]. Interestingly, among essential CVD medicines, the MPR for the MSG was even higher than that for LPG, suggesting that costlier brands of generic medicines are probably more prescribed and/or dispensed than available cheaper generics, potentially resulting in speculation by retailers based on demand. The limited availability of EMs in the public-sector hospitals in LMICs is usually caused by deficiencies of the supply chain, suboptimal coordination in the distribution chain and inadequate funding; however, it may also be driven by the belief that cheaper means lower quality, leading to consumer’s preference for higher priced medicines [25]. Indeed, a high proportion of lay people, doctors and pharmacists have negative perceptions regarding the quality, efficacy and safety of generic medicines, which are deemed as of inferior quality than branded medicines [26].

While understanding that private-sector prices have to take into account additional costs (such as taxes, tariffs, margins) in the pharmaceutical supply chain [7] compared to prices in the public sector, the differences we found represent an uneven application of statutory profit and cost ceilings (against the local law that stablishes profit mark-ups) and suggests poor local market control by the regulatory bodies. An earlier economic evaluation of medicines price in urban Mozambique comparing the lowest priced version of drugs in both sectors had concluded that local mark-ups determine a large proportion of the final price of medicines sold and showed a clear segmentation of the market [22]; in Maputo low-cost retailers acted in peripheral neighborhoods while in central Maputo pharmacies prices are higher, with price discrimination depending on the aspect and location of the pharmacy, as well as the social class and purchase capacity of customers [22].

The present study also found that the mean availability of diagnostic tests and devices in the day of survey was much higher in private (89.5% and 91.7%) than public hospitals (55.6% and 58.3%), respectively. Among public hospitals, where patients do not pay for diagnostics, we have found that less than 35% had some essential tests such as troponin, natremia, kalemia, ECG and echocardiogram available at the time of visit and none had LDL-Cholesterol and HbgA1C tests available. In private sector, where the availability was high (above 90% for both tests and devices), high prices were charged.

To purchase a monthly supply of most CVD EMs or to perform an annual risk monitoring without purchasing any medicine, a lowest paid unskilled worker would have to spend more than one day’s wage per month. The expenditure would increase substantially with increased complexity of evaluations and treatments, reaching more than 15 days’ wage per month if secondary prevention was needed, much higher than what is reported elsewhere [27].

Low availability, high prices and poor affordability represent major barriers to access to medicines [28,29,30,31], a major component of access to health care. Surveys deployed worldwide have highlighted disparities in drug availability and prices by region, therapeutic category and sector [30,32]. By including a comprehensive range of evidence-based CVD medicines (including paediatric formulations) the present study brings a more complete evaluation of access to essential CVD medicines in the targeted setting. The addition of basic diagnostic tests and devices, constitutes a scaling up of this standard methodology that can be used for comparisons between regions and countries, and will hopefully promote equitable CVD care. Even so, some limitations of our study should be acknowledged. Firstly, availability and prices were evaluated for the tailored list of medicines that was generated for this study, and comparisons with previous studies should take this into account. Secondly, the variations of availability that occur over time are not captured with this methodology, which used data referring to the day of survey. Finally, for the affordability analysis, one should consider that the lowest paid government worker earns more than the minimum wage in the general population, and thus our results may in fact be a conservative measure of affordability. Nevertheless, we purposefully used this standardized WHO/HAI methodology to allow for future comparisons within the same country, as well as with other countries.

## Conclusion

Access to CV care is low in public and private sectors of Maputo city, as shown by the overall low availability, high prices and poor affordability to EMs and diagnostics that were found. Considering the EML CV medicines were among the most expensive, showed the highest disproportion between IRP and cost at pharmacy, and were much less available. Unavailability of the surveyed diagnostic tests and devices in the public sector determined high OOP costs. The expanded EML should inform policies to improve access to CV care in Mozambique.

## Additional File

The additional file for this article can be found as follows:

10.5334/gh.1186.s1 Appendix. Tables S1 and S2.
