# Supplementary material for: Access to Essential Medicines and Diagnostic Tests for Cardiovascular Diseases in Maputo City, Mozambique
Source: Glob Heart. 2023 Feb 28;18(1):8. doi: 10.5334/gh.1186 (PMC9983496; doi:10.5334/gh.1186)
Supplement: Appendix. — Tables S1 and S2. [file gh-18-1-1186-s1.pdf]

APPENDIX

Table S1. List of medicines surveyed in Maputo city, Mozambique.

	Medicine	Strength	Dosage form/unit
	**Core Essential Medicines**		
1	Amitriptyline	25 mg	Tab/cap
2	Amoxicillin	500 mg	Tab/cap
3	Atenolol	50 mg	Tab/cap
4	Captopril	25 mg	Tab/cap
5	Ceftriaxone Inj	1 g/vial	Vial
6	Ciprofloxacine	500 mg	Tab/cap
7	Co-trimoxazole suspension	8+40 mg/ml	ml
8	Diazepam	5 mg	Tab/cap
9	Diclofenac	50 mg	Tab/cap
10	Glibenclamide	5 mg	Tab/cap
11	Omeprazole	20 mg	Tab/cap
12	Paracetamol suspension	24 mg/ml	ml
13	Salbutamol inhaler	100 mcg/dose	dose
14	Simvastatin	20 mg	Tab/cap
	**CVD Essential Medicines**		
1	Bisoprolol	5 mg	Tab/cap
2	Glyceryl trinitrate (sublingual)	0.5 mg	Tab/cap
3	Isosorbid dinitrate (sublingual)	5 mg	Tab/cap
4	Digoxin solution	0.05 mg/ml	Tab/cap
5	Lidocaine	20mg/ml in 5 ml vial	Vial
6	Verapamil (hydrochloride)	40 mg	Tab/cap
7	Amiodarone	100 mg	Tab/cap
	Amiodarone	50 mg/ml, 3 ml Ampoule	Ampoule
8	Amlodipine maleate	5 mg	Tab/cap
9	Elanapril (as hydrogen maleate)	5 mg	Tab/cap
10	Hydralazine, powder for injection	20 mg	Ampoule
	Hydralazine	25 mg	Tab/cap
11	Hydrochlorothiazide	25 mg	Tab/cap
12	Methyldopa	250 mg	Tab/cap
13	Losartan	50 mg	Tab/cap
14	Furosemide	40 mg	Tab/cap
		10 mg/ml, 2 ml ampoule	Ampoule
		20 mg/5ml	Vial
15	Spironolactone	25 mg	Tab/cap
16	Dopamine	40 mg/ml in 5 ml	Vial
17	Acetylsalicylic acid	100 mg	Tab/cap
18	Clopidogrel	75 mg	Tab/cap
19	Streptokinase	1.5 million IU	Vial
20	Glicazide (controlled release)	60 mg	Tab/cap
21	Glucagon	1mg/ml	Injection
22	Metformin (hydrochloride)	500 mg	Tab/cap

23	Benzathine benzyl penicillin		Vial
24	Heparin	5000 IU/ml	Vial
25	Warfarin	5 mg	Tab/cap
26	Morphine	10 mg/ml	Ampoule
27	Phenoxyl Methyl penicillin	250 mg	Tab/cap
28	Erythromycin	250 mg	Tab/cap
29	Nifedipine retard	30 mg	Tab/cap
30	Soluble insulin	100 IU/ml	Vial
31	Adrenaline	1 mg/ml	Vial
32	Sodium Nitroprusside (powder)	50 mg	Ampoule
33	Metoclopramide (hydrochloride)	5 mg/ml in 2ml-ampoule	Ampoule
	Metoclopramide	5 mg/5ml	Oral susp
	Metoclopramide (hydrochloride)	10 mg	Tab/cap
34	Omeprazole	20 mg	Tab/cap
35	Digoxin	0.25 mg	Tab/cap
36	Phenoxymethyl penicillin	500 mg	Tab/cap
	**Diagnostic tests**	**Diagnostic devices**	
1	Glycaemia	Thermometer	
2	Creatinine	Electrocardiograph	
3	Urea	Weighing scale/machine	
4	Total cholesterol	Sphygmomanometer	
5	HDL cholesterol	Stethoscope	
6	LDL cholesterol	Pulse oximeter	
7	Triglyceride	Space for inhalers	
8	Proteinuria	Glucometer	
9	Natremia	Peak flow meter	
10	Kalemia	Blood glucose test strips	
11	HbA1c	Urine protein test strips	
12	Uric acid	Urine ketone test strips	
13	Full blood count	Nebulizer	
14	ESR	Troponin test strips	
15	Troponin	Urine albuminuria test strips	
16	ASO	Tuning fork	
17	Electrocardiogram	Defibrillator	
18	Echocardiogram		
19	Chest X-ray (radiography facility)		

ASO, Antistreptolysin O; ESR, erythrocyte sedimentation rate.

Table S2. MPR of surveyed essential medicines in the private retail sector in Maputo city, Mozambique.

**Medicine**	**MSH Reference Price (2015 USD)**	**Medicine Type**	**Median Consumer Price**		**Median Price Ratio (with respect to MSH reference Price)**
			**In USD**	**In MTN**	
WHO Core essential Medicines					
Amitriptyline 25 mg cap/tab	0.0084	Most sold generic	0.0410	2.5000	4.88
		Lowest priced generic	0.0410	2.5000	4.88
Amoxicillin 500 mg cap/tab	0.0299	Most sold generic	0.0574	3.5000	1.92
		Lowest priced generic	0.0574	3.5000	1.92
Atenolol 50 mg cap/tab	0.0107	Most sold generic	0.1393	8.5000	13.02
		Lowest priced generic	0.0820	5.000	7.66
Captopril 25 mg cap/tab	0.0246	Most sold generic	0.0410	2.5000	1.67
		Lowest priced generic	0.0410	2.5000	1.67
Ceftriaxone Inj 1 g/vial vial	0.3980	Most sold generic	1.9474	118.79	4.89
		Lowest priced generic	1.9474	118.79	4.89
Ciprofloxacine 500 mg cap/tab	0.0373	Most sold generic	0.0984	6.0000	2.64
		Lowest priced generic	0.0920	5.6100	2.47
Co-trimoxazole suspension 8+40 mg/ml mililitres	0.0048	Most sold generic	0.0137	0.8333	2.85
		Lowest priced generic	0.0131	0.8000	2.73
Diazepam 5mg cap/tab	0.0096	Most sold generic	0.1057	6.4500	11.01
		Lowest priced generic	0.1057	6.4500	11.01
Diclofenac 50 mg cap/tab	0.0045	Most sold generic	0.0128	0.7791	2.84
		Lowest priced generic	0.0121	0.7396	2.69
Glibenclamide 5mg cap/tab	0.0057	Most sold generic	0.0164	1.0000	2.88
		Lowest priced generic	0.0164	1.0000	2.88
Omeprazole 20 mg cap/tab	0.0141	Most sold generic	0.0495	3.0205	3.51
		Lowest priced generic	0.0451	2.7500	3.20
Paracetamol suspension 24mg/ml millilitre	0.0052	Most sold generic	0.0131	0.8000	2.52
		Lowest priced generic	0.0111	0.6800	2.14
Salbutamol inhaler 100 mcg/dose dose	0.0092	Most sold generic	0.0274	1.6700	2.98
		Lowest priced generic	0.0274	1.6700	2.98
Simvastatin 20 mg tab/cap	0.0163	Most sold generic	0.1619	9.8750	9.93
		Lowest priced generic	0.1619	9.8750	9.93
**CVD medicines**					
Bisoprolol 5mg tab/capsule	0.0912	Most sold generic	0.4391	26.7857	4.81
		Lowest priced generic	0.0891	5.4333	0.98
Glyceryl trinitrate Sublingual tablet 500 mcg	0.0771	Most sold generic	N/A		
		Lowest priced generic			
Isosorbid Dinitrate Sublingual tablet 5mg	0.0701	Most sold generic	N/A		
		Lowest priced generic			
Digoxin 0.05 mg/ml solution, milliliter #	0.2506	Most sold generic	N/A		
		Lowest priced generic			
Lidocaine 20mg/ml in 5 ml Vial	N/A	Most sold generic	0.0823*	5.0200*	--
		Lowest priced generic	0.0823*	5.0200*	--
Verapamil	0.0470	Most sold generic	N/A		

(hydrocholoride) 40 mg tab		Lowest priced generic			
Amiodarone Tab 100mg		Most sold generic	N/A		
		Lowest priced generic			
Amiodarone 50mg/ml in 3 ml Ampoule	0.3330	Most sold generic	N/A		
	0.3330	Lowest priced generic			
Amlodipine maleate 5mg Tab	0.0061	Most sold generic	0.1219	7.4333	19.98
		Lowest priced generic	0.1210	7.3833	19.84
Enalapril (as hydrogen maleate) 5 mg Tab	0.0104	Most sold generic	0.1322	8.0667	12.72
		Lowest priced generic	0.1322	8.0667	12.72
Hydralazine, 20 mg Powder for Inj. (hydrocholoride) in Ampoule	3.8578	Most sold generic	N/A		
		Lowest priced generic			
Hydralazine, 25mg Tab/cap	0.0407	Most sold generic	N/A		
		Lowest priced generic			
Hydrochlorothiazide, 25 mg Tab/cap	0.0043	Most sold generic	0.0656*	4.000*	--
		Lowest priced generic	0.0656*	4.000*	--
Methyldopa, 250 mg Tab/cap	0.0324	Most sold generic	0.0795	4.8495	2.45
		Lowest priced generic	0.0795	4.8495	2.45
Losartan, 50 mg Tab/cap	0.1151	Most sold generic	0.2363	14.4167	2.05
		Lowest priced generic	0.2363	14.4167	2.05
Furosemide, 40mg Tab/cap	0.0061	Most sold generic	0.0195	1.1920	3.20
		Lowest priced generic	0.0195	1.1920	3.20
Furosemide, 10 mg/ml in 2- ml ampoule	0.0520	Most sold generic	N/A		
		Lowest priced generic			
Furosemide, 20 mg/5 ml		Most sold generic	N/A		
		Lowest priced generic			
Spironolactone, 25 mg Tab/Cap	0.0405	Most sold generic	0.2346	14.3100	5.79
		Lowest priced generic	0.2346	14.3100	5.79
Dopamine Inj, 40 mg/ml in 5 ml Vial	0.1123	Most sold generic	N/A		
		Lowest priced generic			
Acetylsalicylic acid, 100 mg Tab/cap	0.0021	Most sold generic	0.1220	7.4417	58.09
		Lowest priced generic	0.1220	7.4417	58.09
Clopidogrel, 75 mg Tab/cap	0.1011	Most sold generic	0.5834	35.5893	5.77
		Lowest priced generic	0.5834	35.5893	5.77
Streptokinase (powder for Inj), 1.5 million IU in Vial	38.5596	Most sold generic	N/A		
		Lowest priced generic			
Gliclazide (controlled release) 60 mg Tab		Most sold generic	0.1500	9.1500	6.76
		Lowest priced generic	0.1500	9.1500	6.76
Glucagon, 1 mg/ml Inj (vial)	22.3000	Most sold generic	N/A		
	22.3000	Lowest priced generic			
Metformin (hydrocholoride), 500 mg Tab/cap	0.015	Most sold generic	0.1093	6.6667	7.29
		Lowest priced generic	0.0328	2.0000	2.19
Benzathine Benzyl	0.2619	Most sold generic	1.5492	94.5000	5.93

penicillin, 2.4 mega units Inj (per vial)		Lowest priced generic	1.5492	94.5000	5.93
Heparin, 5000 IU/ml Inj (per ml)	0.7122	Most sold generic	N/A		
		Lowest priced generic			
Warfarin 5mg Tab/cap	0.0436	Most sold generic	0.0896	5.4667	2.06
		Lowest priced generic	0.0896	5.4667	2.06
Morphine, Injection 10mg/ml, in 1ml ampoule	0.5315	Most sold generic	N/A		
		Lowest priced generic			
Phenoxymethyl penicillin, 250 mg Tab/cap	00176	Most sold generic	N/A		
		Lowest priced generic			
Erythromycin, 250 mg Tab/cap	0.0372	Most sold generic	N/A		
		Lowest priced generic			
Nifedipine retard, 30 mg Tab/Cap	0.0272	Most sold generic	0.0689	5.3000	3.19
		Lowest priced generic	0.8197	5.0000	3.01
Soluble insulin 100 IU, per ml	0.5657	Most sold generic	N/A	N/A	
		Lowest priced generic	N/A	N/A	
Adreline/Epinephrine, 1mg/ml Inj	0.1753	Most sold generic	N/A		
		Lowest priced generic			
Sodium nitroprusside, Powder for infusion: 50 mg in ampoule	N/A	Most sold generic	N/A		
		Lowest priced generic			
Metoclopramide, Injection: 5 mg (hydrochloride)/mL in 2- mL ampoule (per ml)	0.0072	Most sold generic	1.9016*	116.0000*	--
		Lowest priced generic	1.9016*	116.0000*	--
Metoclopramide, Oral liquid: 5 mg/5 mL, milliliter	0.0072	Most sold generic	0.0025*	0.7550*	--
		Lowest priced generic	0.0025*	0.7550*	--
Metoclopramide, Solid oral: 10 mg (hydrochloride)	0.0066	Most sold generic	0.0328	2.0000	4.97
		Lowest priced generic	0.0328	2.0000	4.97
Digoxin 0.25mg, solid oral	0.0101	Most sold generic	0.1142	6.9667	11.31
		Lowest priced generic	0.1142	6.9667	11.31
Phenoxymethyl penicillin, 500 mg Tab/cap	0.0243	Most sold generic	0.0984	6.000	4.05
		Lowest priced generic	0.0984	6.000	4.05
Metoclopramide, Injection: 10 mg (hydrochloride)/mL in 2- mL ampoule		Most sold generic			
		Lowest priced generic			
N/A = not available in surveyed facilities; * Less than four price data points were available so MPR was not calculated.					
